# Relative colon wall attenuation – distinguishing ischemia from other colon wall changes

**DOI:** 10.1186/s12880-026-02713-5

**Published:** 2026-08-21

**Authors:** Jonas Oppenheimer, Sophia Lüken, Christian Schineis, Elena Sonnenberg, Hans Tepe, Ingo Steffen, Thomas Elgeti, Lars-Arne Schaafs

**Affiliations:** 1 Department of Radiology, Charité-Universitätsmedizin Berlin, Corporate Member of Freie Universität Berlin and Humboldt-Universität zu Berlin, Berlin, Germany; 2 Department of General and Abdominal Surgery, Campus Benjamin Franklin, Charité-Universitätsmedizin Berlin, Corporate Member of Freie Universität Berlin and Humboldt-Universität zu Berlin, Berlin, Germany; 3 Department of Gastroenterology, Infectious Diseases and Rheumatology, Campus Benjamin Franklin, Charité-Universitätsmedizin Berlin, Corporate Member of Freie Universität Berlin and Humboldt-Universität zu Berlin, Berlin, Germany

**Keywords:** Contrast enhanced CT, Mesenteric ischemia, Colon ischemia, Bowel ischemia

## Abstract

**Background:**

Differentiation of bowel wall changes between ischemia and other causes in CT imaging can prove challenging, particularly in colon wall. Therefore, measurement of relative colon wall attenuation and contrast uptake were evaluated for aiding in diagnosis of colon ischemia.

**Methods:**

CTs with inquiry for bowel ischemia were retrospectively identified. Cases involving colon ischemia or colon wall changes of other etiologies were included. Three regions of interest (ROIs) were placed in representative areas of colon wall with and without evident changes, in arterial and venous phase images, respectively. Means of the ROIs were used to calculate the relative bowel enhancement between affected and unaffected wall in each contrast phase, as well as the contrast uptake of the wall between phases. Relative enhancement and contrast uptake were compared between patients with and without ischemia. A subset of cases was reanalyzed for intra- and inter-reader comparison.

**Results:**

31 patients with and 56 patients without ischemia were included. Relative enhancement of affected to unaffected colon wall was  -51.2% in arterial phase and  -44.6% in venous phase images for patients with ischemia. Relative enhancement was significantly higher in patients without ischemia at -0.5% in both phases (*p* < 0.001). There was no significant difference in contrast uptake of affected bowel (40.6% vs. 39.8% respectively, *p* = 0.67). ROC curves revealed a maximum Youden-Index at a cut-off of -37.4% (AUC: 0.818) in arterial phase to differentiate between groups. Intra-reader comparison showed a good, and inter-reader a moderate, correlation in a subset.

**Conclusions:**

Objective measurement of relative colon wall attenuation between affected and unaffected areas can be an additional imaging feature to aid in identifying ischemic colon.

**Supplementary Information:**

The online version contains supplementary material available at 10.1186/s12880-026-02713-5.

## Background

Bowel ischemia is a can-not-miss diagnosis with a potentially severe morbidity and mortality. While relatively rare, with incidence rates reported between 5.3 and 8.7 per 100,000 people per year [1, 2], studies have shown a high mortality for acute mesenteric ischemia (AMI) of up to 72% [3]. Risk for mesenteric ischemia increases sharply with age, with AMI becoming a common cause of acute abdomen in those over 75 [4]. Due to a high overlap of symptoms and laboratory tests with other etiologies of abdominal pain [1], contrast-enhanced CT imaging plays a central role in the further diagnostic workup [5, 6].

Where there is no clear evidence of a vascular obstruction in arterial phase (AP) or venous phase (VP) imaging, diagnosis is based on the evidence of changes to the bowel wall and the mesentery. CT imaging shows an overall high diagnostic accuracy and several radiologic signs have been identified for the diagnosis of ischemia, including primarily a decreased bowel wall enhancement [7, 8]. However detection and definition of hypoenhancement is highly subjective and differentiating changes in the bowel wall and mesentery between ischemia and other causes, such as inflammation, remains difficult [9–12].Research into objective bowel wall enhancement is limited [13]. Changes of the large bowel may be particularly challenging to correctly diagnose in CT imaging [14]. While a gold-standard diagnostic colonoscopy may be accessible, a prior risk stratification by CT can lead to better clinical outcomes [15], and colonic involvement in ischemia has shown a significant effect on morbidity and mortality [16].

This study aims to objectively compare changes in colon wall attenuation in patients with confirmed colon ischemia and those with other diagnoses showing evident colon wall changes in CT, in order to establish whether relative bowel wall attenuation can be used as an additional imaging-based marker to identify bowel ischemia.

## Methods

### Patient selection

The local institutional review board approved this single-center, explorative, retrospective study (EA2/147/24). The study was designed in accordance with STARD recommendations where possible [17], the STARD checklist is included in Appendix 1. CTs of patients with inquiry for bowel ischemia were identified by a full-text search from a tertiary-level clinic’s radiological information system. Reports of CT scans of the abdomen were identified by the key terms “bowel ischemia”, “mesenteric ischemia”, and “ischemia”. Full-text screening of the written reports was performed to discern cases incorrectly identified. Duplicate cases of the same patient in the same clinical stay were excluded. In a two-year period from May 2022 to May 2024, 1129 exam reports were screened, and 646 sequential exams were included for preliminary review. Patient files were reviewed to identify cases of colonic ischemia diagnosed by the gold standard (colonoscopy or laparoscopy) during the same clinical stay as the CT exam. Additionally, all cases where either ischemia was ruled out by the gold standard or no gold standard testing was performed, as ischemia was deemed unlikely, were reviewed for imaging changes of the colon wall and included, if evident. Cases where clear CT evidence of bowel necrosis was diagnosed, but further workup was deferred by the patient for palliative care, were excluded. From both groups, cases were excluded if there was not at least an arterial and venous phase scan performed at initial presentation. Lastly, cases were excluded where there was no unaffected bowel for comparison.

### Patient characteristics

Patient sex and age were noted. An estimated Body-Mass-Index was calculated by weight and height from the CT-dose protocol, where available. The lactate level measured by blood gas analysis closest to the CT-scan was noted. Missing values were excluded from calculated averages. The referring clinic was noted (emergency department (ED), ward, intensive care unit (ICU)), as well as the length of the hospital after the CT, including days spent in the ICU. “Time to symptom onset” was determined where possible and categorized into (1) acute onset, (2) onset within 24 h, (3) within 48 h, (4) within 72 h, (5) within one week, (6) within one month, and (7) chronic symptoms. For patients with surgically confirmed ischemia, time between the CT-scan and the beginning of the surgical intervention was noted, rounded to the nearest hour. Clinical patient outcomes were categorized by (1) direct discharge, (2) ward stay, (3) ICU stay, (4) death within 24 h after CT scan, (5) death within 72 h, (6) death within one week, and (7) death within any period in the same hospital stay. For cases where no ischemia was present, the best alternative diagnosis was broadly categorized.

### CT acquisition

All included patients underwent a CT of the abdomen and pelvis in both arterial and venous phases, with automated aortic bolus tracking for arterial triggering and a post-injection delay of 80 s for VP. Additionally acquired contrast phases were not used for further analysis. Exams were performed on a Canon Aquilion PRIME or Aquilion ONE (Canon Medical System Corporation, Otawara, Japan) with patient-adapted automatic dose modulation, using a kVp of 100–120 and adaptive Amperage. 80–120 ml of Iodinated contrast media (Xenetix 350, Guerbet SA, Villepinte, France, or Imeron 400, Bracco S.p.A., Milan, Italy) was administered intravenously at a standard rate of 4 ml/s, adjusted to patient characteristics and the scanning protocol as necessary. Thin (0.5–1 mm) and thick-slice (5 mm) axial reconstructions, as well as coronal and sagittal reconstructions in the vendor-standard soft tissue kernel, were provided for each exam.

### Image analysis

**Fig. 1:**
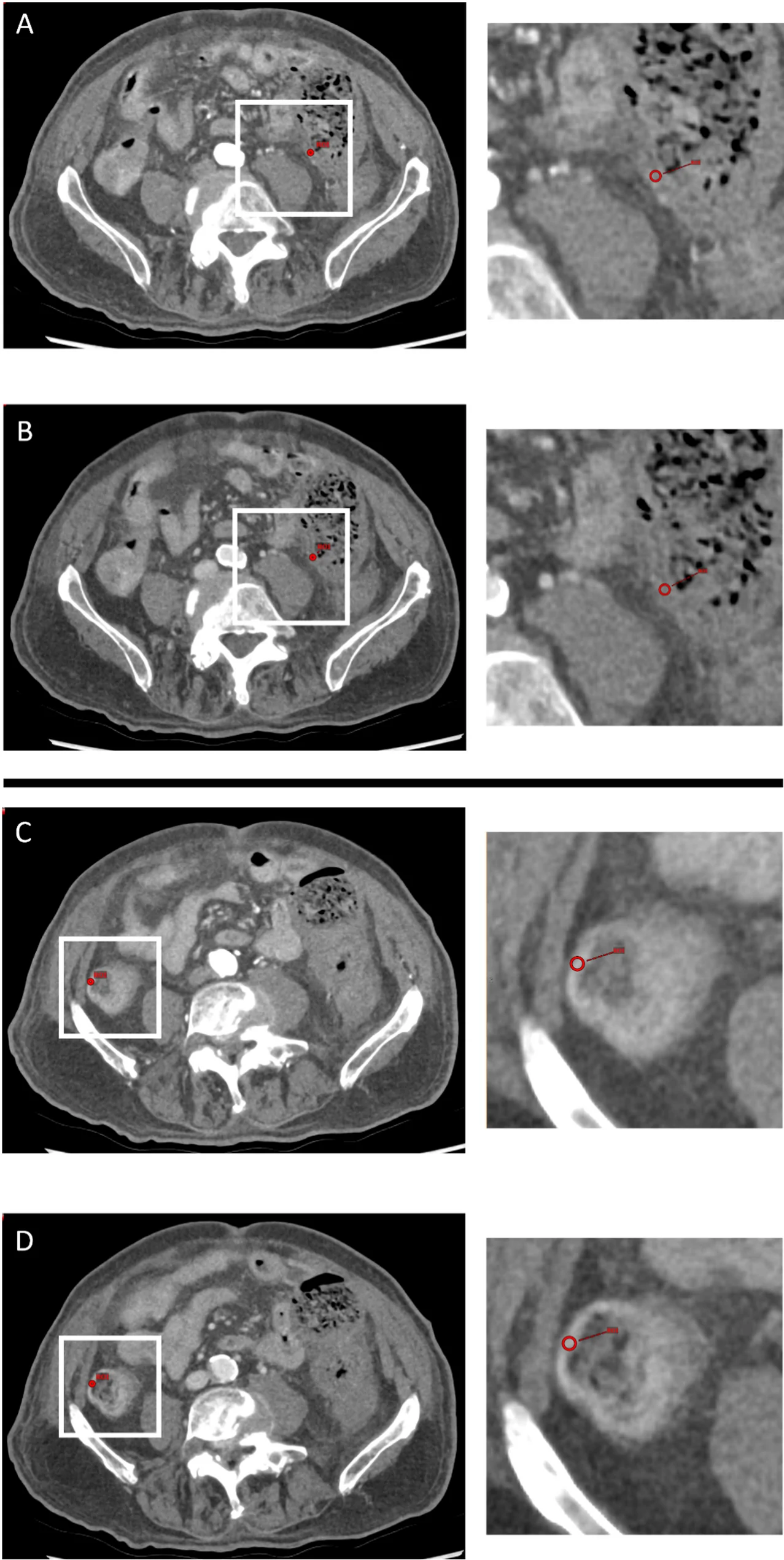
**A**-**D**: Analysis of bowel wall enhancement, placement of region of interest (ROI). Axial CT images of the abdomen with full field of view and zoomed selections (white box) of relevant bowel segments. 3 mm regions of interest (ROI, red) were placed in affected (**A**, **B**) and unaffected (**C**, **D**) bowel wall with identical placement in arterial (**A**, **C**) and venous (**B**, **D**) phase images

Each CT exam was reviewed by a single reviewer with three years of experience in abdominal radiology (J.O.), to define areas of affected and unaffected colon wall, correlating with surgical and endoscopy reports, where available. Image Analysis was performed with Visage 7.1.19 (Visage Imaging, Berlin, Germany). Three regions of interest (ROI) with a diameter of 3 mm were manually placed in the affected and unaffected areas each. A diameter of 3 mm was selected as a practical size to include as large an area of the wall as possible, without being too large to place within the wall. Sections of the wall with a thickness of at least 3 mm were chosen and each ROI was placed as centrally into the bowel wall as possible in regard to the overall thickness. Placement was carefully performed to avoid any strongly contrasted vessels, luminal bowel contents, intraluminal or intramural air, surrounding fatty tissue, as well as areas with strong image artifacts. Each ROI was placed in a separate slice in axial images of thin-slice reconstructions and was positioned at the same slice in both phases. As far as possible, the ROI was placed in the identical location of the bowel wall in both phases; however, due to slight changes in bowel position resulting from normal peristalsis, exact matching was not always possible. Average attenuation in Hounsfield units (HU) and standard deviation of the attenuation for each ROI were noted. An example of ROI placement is presented in Fig. 1. For intra-reader comparison a subset of twenty cases each with and without ischemia was analyzed by the same reviewer four months after completion of the initial analysis, blinded to the exam diagnosis and previous measurements. Ten consecutive cases each were analyzed by a second reviewer (S.L.), with three years of experience in abdominal radiology, for inter-reader comparison.

The thickness of the bowel wall was measured at three representative locations in affected and unaffected segments, and means were calculated respectively. Additionally, presence of pneumatosis of the bowel wall, mesenteric fat stranding (not present/present/not discernable due to high volume ascites), ascites (none/low volume/moderate/high volume), bowel lumen distension (none/moderate/ileus), parenchymal organ ischemia (none/singular organ moderately affected/singular organ severely affected/multiple organs affected) and free intraabdominal air (not present/present/present likely due to prior surgery/open abdomen) was noted.

### Statistical analysis

Due to the exploratory nature of the study, no sample size estimation was performed. Averages of the four sets of three ROIs collected for each patient were calculated. The relative attenuation in percent for affected to unaffected bowel wall was calculated for arterial and venous phases for each patient, as well as the relative attenuation of affected and unaffected segments between arterial and venous phases. Calculations are shown in Table 1. A negative relative attenuation indicates a hypoenhancement of the affected bowel wall to unaffected areas.

**Table 1:** Calculations of relative enhancement and contrast uptake

Relative attenuation, arterial phase	$\:\frac{HUaffected\:bowel-HUunaffected\:bowel}{HUunaffected\:bowel}\times\:100\%$
Relative attenuation, venous phase	$\:\frac{HUaffected\:bowel-HUunaffected\:bowel}{HUunaffected\:bowel}\times\:100\%$
Relative contrast uptake, affected segment	$\:\frac{HUvenous\:phase-HUarterial\:phase}{HUarterial\:phase}\times\:100\%$
Relative contrast uptake, unaffected segment	$\:\frac{HUvenous\:phase-HUarterial\:phase}{HUarterial\:phase}\times\:100\%$

Average standard deviation (±) was calculated for each of the three ROIs by calculating the square root of the average of the variances. Reported average standard deviations for the entire group are presented as arithmetic means.

Means of relative attenuation were compared between patients with ischemia and those with other causes of bowel wall changes in arterial and venous phases, as well as the relative contrast uptake of affected segments. The distribution of data was analyzed using histograms and Shapiro-Wilk tests. Means were compared using an independent one-sided t-test for normally distributed data or a Mann-Whitney U test for non-normally distributed data. One-sided testing was justified by the expected overall substantially lower contrast in ischemia compared to other causes of bowel-wall changes. Averages are displayed with the standard deviation (±). Receiver operating characteristic (ROC) curves of comparisons were calculated to discriminate between patients with and without ischemia, using the Youden index to define a cut-off value. The intra- and inter-reader agreement for the subset of cases was calculated using the average measures Intraclass Correlation Coefficient (ICC). Subgroup analysis was performed between different etiologies of ischemic (acute arterial occlusion, non-occlusive mesenteric ischemia (NOMI), chronic ischemia) and non-ischemic (infectious, malignant, other) bowel wall changes, as well as outcome based in the ischemia group, comparing patients with ischemia that survived the hospital stay to those that died during the stay. The threshold of significance was initially set at *p* < 0.05, with a stepwise correction for multiple testing using the Holm-Bonferroni method. Tests where the p-value surpasses the adjusted threshold are declared. Statistical analysis was performed with Microsoft Excel Office 365 Version 2508 (Microsoft Corporation, Redmond, WA, USA) and IBM SPSS Statistics 30.0 (IBM Corporation, Armonk, NY, USA).

## Results

### Patient characteristics

**Fig. 2:**
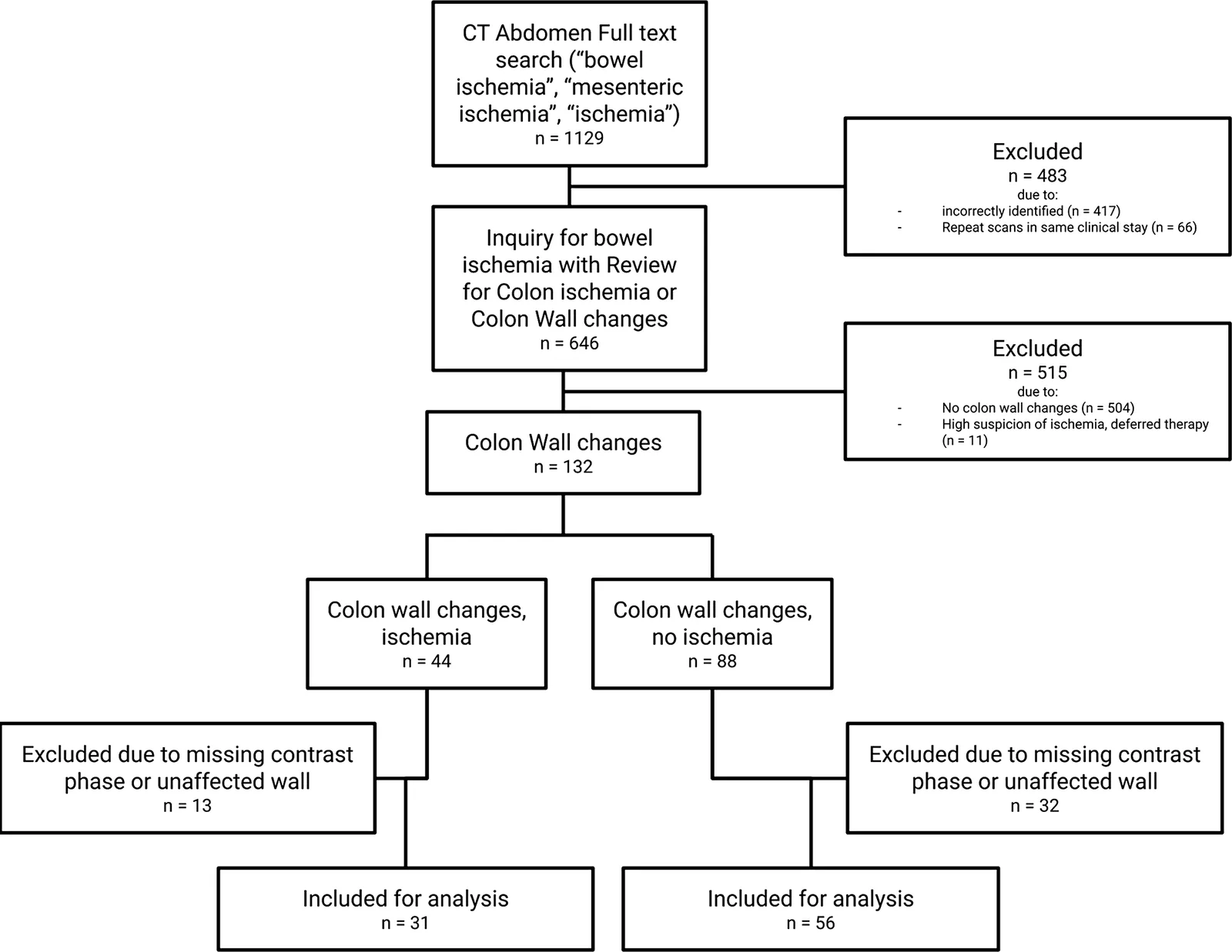
Flowchart displaying the patient selection and final groups included for analysis with and without colon wall ischemia

44 patients with proven colon ischemia were identified, three cases were excluded due to a missing contrast phase, ten cases were excluded as no unaffected bowel wall was discernible for comparison, resulting in 31 cases in the ischemia group. 88 cases with colon wall changes, but no ischemia were identified. Ten cases were excluded due to missing contrast phases and a further 22 cases were excluded due to missing comparable healthy bowel wall, resulting in 56 cases included in the non-ischemia group. The selection of cases is described in detail in Fig. 2. The average age in the ischemia group (74.9 years) was higher than in the non-ischemia group (68.0 years), with a larger proportion of females (58.1% vs. 44.6%). The largest group of patients (13, 41.9%) with ischemia was referred by the ICU; in the non-ischemia group, a majority was referred by the ED (37, 66.1%). Mortality during the same hospital stay as initial CT imaging was higher in the ischemia group (41.9% vs. 14.3%). Relevant patient characteristics are shown in Table 2.

Ischemia was confirmed in patients by colonoscopy alone in 13 cases, by surgery alone in 14 cases, and concordantly in four cases. For patients with a surgical intervention, average time between CT-scan and surgery was 7.9 h (range: 2–26 h, one case with no available data). Bowel was resected in 14 of 18 cases, three cases were ruled as non-salvageable intraoperatively and one case showed only initial ischemia with no necessary intervention. A likely acute arterial occlusion was seen in nine patients (29.0%), an acute venous occlusion was seen in one case (3.1%), twelve cases (38.7%) were ruled as acute NOMI, of which four cases had a chronically occluded inferior mesenteric artery. Eight cases (25.8%) were ruled as chronic ischemic colitis. Mechanical obstruction was ruled as the etiology in the remaining case. Six cases had additionally confirmed intestinal ischemia.

In the non-ischemia group, 57.1% of patients underwent a gold standard procedure ruling out ischemia (14 colonoscopy, 13 surgery, five both). As alternative diagnoses for the bowel wall changes, infectious causes were most common (37 patients, 66.1%), while nine patients had changes due to colon malignancy, and in ten cases the wall changes were ruled as unclear or likely reactive changes.

**Table 2:** Relevant patient statistics

	Ischemia	No ischemia
Average Age	74.9 years (range: 43–95)	68.0 years (range: 40–86)
Male: Female ratio	13:18	31:25
Estimated average BMI *No data available*	25.1 kg/m^2^ 4	30.2 kg/m^2^ 8
Average lactate level (in mg/dl; norm < 16) *No data available*	37.6 4	33.7 5
Referred by		
*ED* *ICU* *Ward*	10 13 8	37 10 9
Time to Onset (No. of patients)		
*Acute* *< 24 h* *< 48 h* *< 72 h* *< 1 week* *< 1 month* *Chronic* *Unknown*	12 6 3 - 4 1 1 4	16 13 9 4 6 3 2 3
Outcome (No. of patients)		
*Direct discharge* *Ward stay (average days)* *ICU stay (average days)* *Death within 24 h after CT* *Death within 72 h after CT* *Death within 1 week after CT* *Death within same hospital stay*	- 8 (17.1 days) 10 (14.7 days) 5 2 3 3	8 25 (15.3 days) 15 (13.5 days) 0 0 3 5

ED – emergency department, ICU – intensive care unit, BMI – body mass index

## Relative attenuation and contrast uptake

**Fig. 3:**
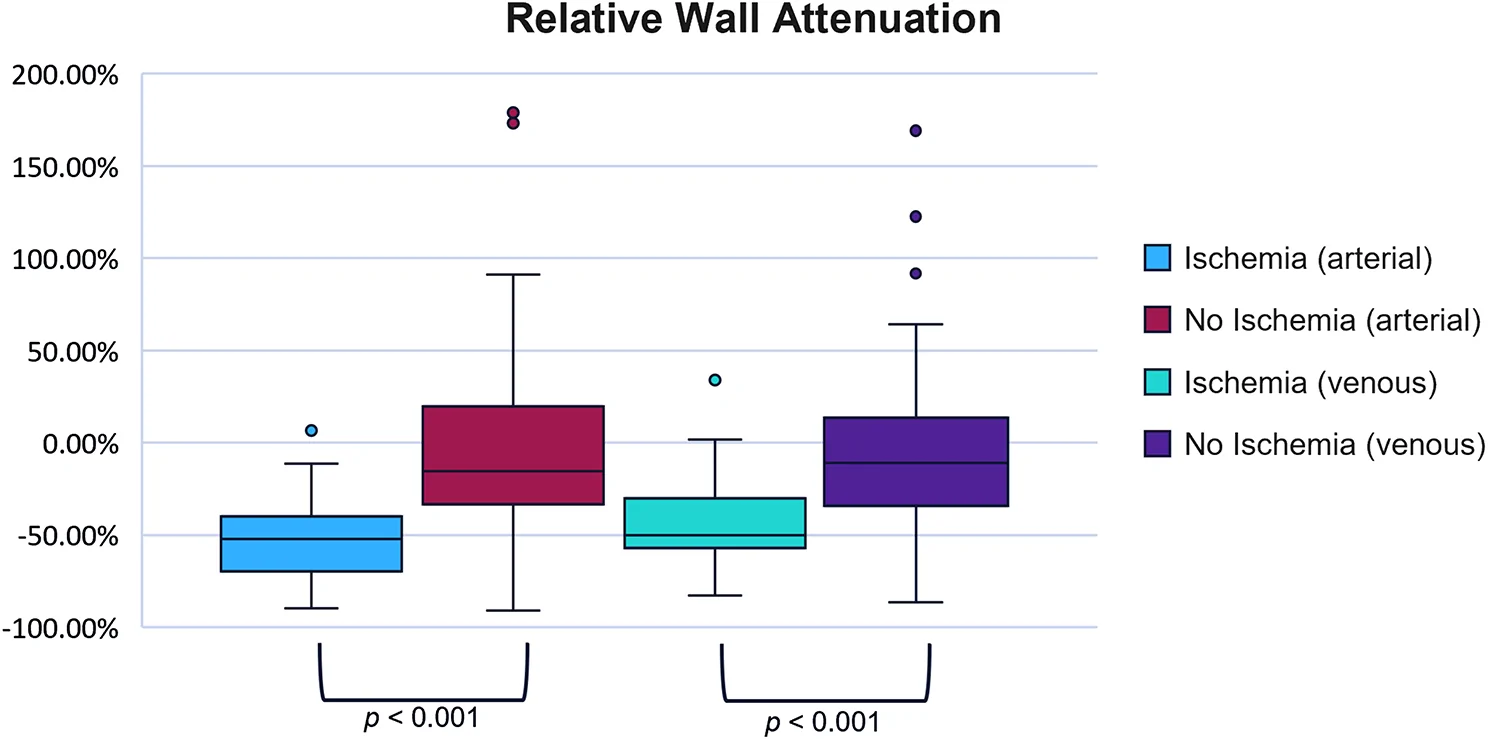
Comparison of relative attenuation of colon wall

Relative attenuation of ischemic bowel to unaffected bowel was 51.2% lower (± 24.2%) than unaffected bowel in the arterial phase and 44.6% lower (± 24.3%) in the venous phase on average. The relative contrast in non-ischemic patients between affected and unaffected bowel was 0.5% lower (t-test, *p* < 0.001) in arterial and 0.5% lower (Mann-Whitney U test, *p* < 0.001) in venous phase; results are compared in Fig. 3. Relative contrast uptake was measured twice in each patient, for affected and unaffected areas respectively, and was averaged within each group. In ischemic patients, relative contrast uptake of affected bowel was 40.6%, higher than in unaffected bowel at 14.5%. Standard deviation of contrast uptake was very high for affected bowel in the ischemia group, as some bowel segments had initial attenuations of only slightly above 0 HU; the effect of the divisor is pronounced, resulting in high outliers. This pattern was similar in the non-ischemia group, with a smaller difference between affected and unaffected bowel, at 39.8% and 27.7%, respectively. Relative contrast uptake of affected bowel was not significantly different between groups (Mann-Whitney U test, *p* = 0.67). Full results are listed in Table 3.

**Fig. 4:**
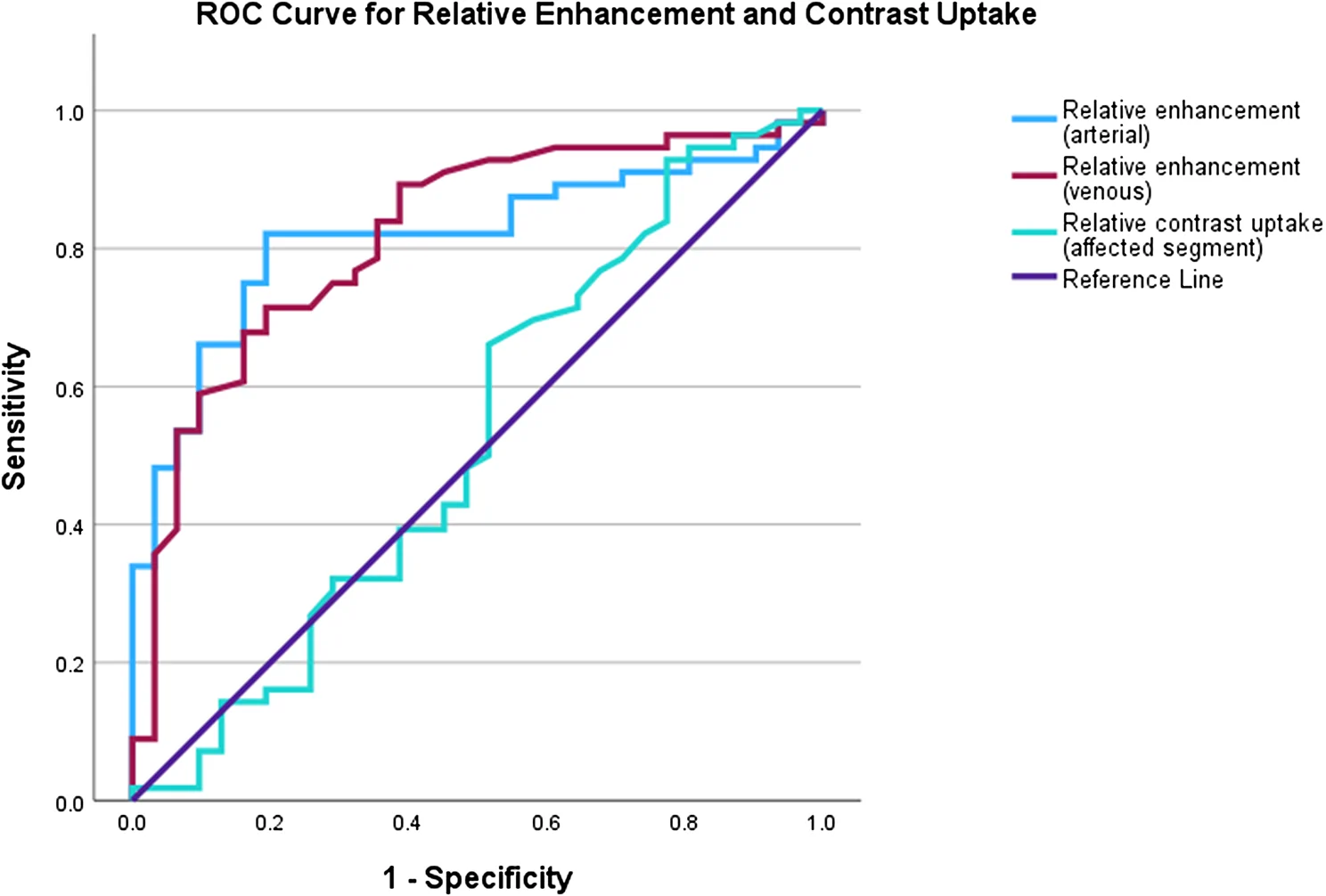
ROC curves for relative attenuation in arterial (blue), venous (red) and relative contrast uptake (green)

Relative attenuation in the arterial and venous phase showed a good area under the curve (AUC) of 0.817 (95% confidence interval: 0.727–0.907) and 0.818 (95% confidence interval: 0.725–0.911), respectively, while relative contrast uptake was a poor differentiator (AUC 0.528, 95% confidence interval: 0.393–0.662). The maximum value of the Youden-Index was reached at a cut-off of -37.4% in arterial phase (sensitivity 82.1%, specificity 80.6%) and of -29.5% in venous phase (sensitivity 71.4%, specificity 80.6%). ROC-curves are shown in Fig. 4.

**Table 3:** Full results of attenuation and contrast uptake of colon wall, means ± standard deviation

	Ischemia	No ischemia
Average attenuation affected bowel wall (arterial phase) in HU *Avg Standard deviation of Measurements*	33.0 (± 15.0; Range: 3.23–59.3) *17.5*	46.4 (± 24.9; Range 3.2–131.3) *15.8*
Average attenuation affected bowel wall (venous phase) in HU *Avg Standard deviation of Measurements*	39.9 (± 16.5; Range 9.8–76.8) *16.2*	56.6 (± 27.1; Range 5.2–147.6) *17.3*
Average attenuation unaffected bowel wall (arterial phase) in HU *Avg Standard deviation of Measurements*	69.2 (± 24.2; Range 27.7–121.0) *21.50*	51.6 (± 21.1; Range 20.4–131.9) *18.8*
Average attenuation unaffected bowel wall (venous phase) in HU *Avg Standard deviation of Measurements*	74.1 (± 20.9; Range 36.9–121.9) *21.4*	61.4 (± 19.4; Range: 29.5–117.9) *19.4*
Average relative enhancement (arterial phase)	-51.2% (± 21.2%; Range − 89.6% – 6.6%)	-0.5% (*p* < 0.001) (± 51.0%; Range: -91.0% – 179.1%)
Average relative enhancement (venous phase)	-44.6% (± 24.3%; Range − 82.7% – 33.9%)	-0.5% (*p* < 0.001) (± 43.6%; Range − 86.3% – 169.3%)
Average relative contrast uptake (affected)	40.6% (± 74.5%; Range − 42.6% – 269.5%)	39.8% (*p* = 0.67) (± 77.9%; Range − 29.4% – 513.0%)
Average relative contrast uptake (unaffected)	14.5% (± 33.7%; Range − 49.7% – 100.3%)	27.7% (± 43.9%; Range − 39.1% – 242.4%)

HU – Hounsfield Units

### Intra- and inter-reader comparison

Intra-reader correlation of relative attenuation in the subset was good for in arterial phase (ICC 0.84; *p* < 0.001) and in venous phase (ICC 0.81; *p* < 0.001). There was a moderate inter-reader correlation in the smaller subset, with a slightly better correlation in venous phase (ICC 0.68; *p* = 0.01) than arterial phase (ICC 0.57; *p* = 0.04, *corrected significance threshold of* *p* < *0.013*). Average relative enhancement in arterial phase of ischemia cases was − 60.0% for reader one and − 48.8% for reader two. A complete comparison between readers is shown in Table 4.

**Table 4:** Comparison of results between readers for a subset of ten patients with and ten without ischemia, means ± standard deviation

	Reader 1		Reader 2	
	Ischemia	No Ischemia	Ischemia	No Ischemia
Average attenuation affected bowel wall (arterial phase) in HU	23.4 (± 10.0)	48.9 (± 25.5)	31.6 (± 18.1)	39.1 (± 22.2)
Average attenuation affected bowel wall (venous phase) in HU	27.3 (± 11.5)	52.2 (± 28.6)	34.9 (± 17.0)	54.7 (± 23.2)
Average attenuation unaffected bowel wall (arterial phase) in HU	59.2 (± 20.2)	53.3 (± 21.7)	64.2 (± 23.2)	42.7 (± 20.9)
Average attenuation unaffected bowel wall (venous phase) in HU	66.9 (± 16.6)	53.1 (± 11.8)	55.6 (± 26.0)	43.2 (± 16.2)
Average relative enhancement (arterial phase)	-60.0% (± 16.5%)	-1.7% (± 62.0%)	-48.8% (± 25.3%)	-0.8% (± 46.2%)
Average relative enhancement (venous phase)	-58.6% (± 17.0%)	0.0% (± 62.3%)	-37.1% (± 28.1%)	40.2% (± 64.1%)
Average relative contrast uptake (affected)	40.3% (± 73.9%)	7.9% (± 25.4%)	7.9% (± 69.7%)	67.9% (± 94.5%)
Average relative contrast uptake (unaffected)	21.3% (± 36.8%)	9.9% (± 29.8%)	1.6% (± 61.6%)	19.5% (± 53.5%)

HU – Hounsfield Units

### Subgroup analysis

Relative hypoenhancement of the bowel wall differed substantially in venous phase in the chronic ischemia subgroup in comparison to cases with acute ischemia due to arterial occlusion or NOMI (-24.2% vs. -49.2% and  -54.8%, respectively), while differences were minimal in the arterial phase. Similarly, when comparing patients with ischemia who survived with those that passed away during the hospital stay, there was a moderate difference in relative hypoenhancement in the venous phase (-37.% vs. -54.2%). Different causes of bowel wall changes in the non-ischemia group showed substantial differences in relative enhancement, with malignant causes showing a marked hyperenhancement on average, while infectious cases showed a slight hypoenhancement and the group with changes of uncertain etiology showed a more profound hypoenhancement. Full results are shown in Table 5.

**Table 5:** Comparison of means between different subgroups with and without ischemia, between different etiologies of colon wall changes and patient outcomes

	Ischemia					No Ischemia		
	Occlusion *(n = 9)*	NOMI *(n = 12)*	Chronic *(n = 8)*	Death *(n = 18)*	Survived *(n = 13)*	Infectious *(n = 37)*	Malignant *(n = 9)*	Other *(n = 10)*
Average relative enhancement (arterial phase)	-48.4%	-49.4%	-48.4%	-51.9%	-50.7%	-8.7%	41.6%	-34.1%
Average relative enhancement (venous phase)	-48.6%	-54.8%	-24.2%	-54.2%	-37.6%	-11.1%	38.0%	-20.6%
Average relative contrast uptake (affected)	31.1%	25.8%	25.6%	40.6%	40.6%	31.5%	25.1%	83.6%
Average relative contrast uptake (unaffected)	23.9%	21.1%	-8.4%	29.5%	3.7%	33.4%	19.6%	13.8%

NOMI – non-occlusive mesenteric ischemia, n – number of patients in subgroup

### Ancillary imaging features

Differences in ancillary imaging features between the groups varied widely. Affected bowel wall was more thickened in both groups, somewhat more prominently in the non-ischemia group (6.2 mm vs. 3.5 mm in the non-ischemia group, 5.2 vs. 3.3 mm in the ischemia group). Fat stranding and ascites were present in the majority of cases in both groups; fat stranding was more common in the non-ischemia group, while a larger percentage of patients had ascites in the ischemia group. Pneumatosis coli was present only present in ischemia cases. Parenchymal organ ischemia was present in 14 ischemia cases and only three non-ischemia cases. A complete comparison of ancillary imaging features is shown in Table 6.

**Table 6:** Comparison of ancillary imaging features between cases with and without colon ischemia, means ± standard deviation

	Ischemia	No Ischemia
Bowel wall thickness, affected segment	5.2 mm (± 2.2, Range 2.0–11.4 mm)	6.2 mm (± 3.4, Range 2.1–20.8 mm)
Bowel wall thickness, unaffected segment	3.3 mm (± 1.1, Range 1.6–6.2 mm)	3.5 mm (± 1.5, Range 1.6–10.0 mm)
Pneumatosis colli	4 (12.9%)	0
Mesenteric fat stranding *Non-diagnostic due to ascites*	19 (61.3%) 2	46 (82.1%) 1
Ascites *Low-volume* *Moderate-volume* *Large-volume*	20 (64.5%) 10 8 2	28 (50.0%) 20 7 1
Ileus Moderate luminal distension	1 (3.2%) 3	7 (12.5%) 4
Parenchymal organ ischemia *One organ (moderate)* *One organ (severe)* *Multiple organs*	14 (45.2%) 1 5 8	3 (5.4%) 1 1 1
Free intraperitoneal air *Due to perforation* *Likely postoperative* *Open abdomen*	7 2 (6.5%) 4 1	10 8 (14.3%) 2 0

## Discussion

This study objectively compared colon wall enhancement in patients with colon ischemia to those with colon wall changes of other etiologies. A significant difference in relative hypoattenuation of bowel wall between ischemic and non-ischemic bowel wall changes was found. Calculating a patient-intrinsic relative enhancement rather than absolute cut-off values mitigates patient effects on overall contrast attenuation, i.e. due to the relationship of contrast dose and bodyweight or cardiac output. This method also factors out potential effects on absolute attenuation that may be present by the use of different scanners or contrast media. Only two cases of ischemia showed a relative hyperattenuation in venous phase, both were classified as chronic ischemia by endoscopy, with chronic ischemia showing an overall more moderate hypoenhancement of the bowel wall in the venous phase. A third case had a relative hyperattenuation in the arterial phase, with endoscopically confirmed mild ischemia after successful revascularization; no surgical resection was required. The range of relative enhancement was wider in the non-ischemia group, in line with the diverse causes of bowel wall changes. Changes are slightly more pronounced in the arterial phase; a cut-off of over 37% hypoehancement showed a robust sensitivity and specificity. This cut-off should be regarded only as a trend and not a fixed boundary, as there is a risk of overfitting onto the current dataset. Further research is needed to validate a diagnostic cut-off in external datasets. Interestingly, the ten non-ischemia cases meeting this threshold had a variety of different differential diagnoses, three cases had a colitis, two with a perforation due to underlying colon malignancy, one case of volvulus, and four cases of reactive edema due to hepatopathy or cardiac failure. When additionally applying the venous cut-off value of over 30% relative hypoenhancement, three cases would be classified as non-ischemic; therefore, a combination of both cut-off values may increase specificity. Notably, relative contrast uptake did not demonstrate significant differences between ischemic bowel wall changes and other etiologies. Contrast uptake of ischemic bowel segments was higher than that of unaffected segments in the same patients, although a large standard deviation may indicate different effects of delayed residual perfusion. Furthermore, the contrast uptake of healthy bowel wall was lower in the ischemia group than in the non-ischemia group, potentially attributable to a larger percentage of severely ill patients.

A variety of radiologic signs to diagnose bowel ischemia have been discussed previously, many of which were in included in this study [6]. Nevertheless, definitive signs with both high sensitivity and specificity, as well as good reproducibility between readers, remain elusive [18]. While decreased bowel wall enhancement is an apparent feature of mesenteric ischemia, diagnosis is primarily based on subjective interpretation, and sensitivity varies, particularly in early stages of ischemia [1, 12, 19]. Furthermore, inter-reader agreement of absent wall enhancement has been shown to be lower in the assessment of the colon wall than of the small intestine [20]. The objectifiable approach demonstrated in this study may mitigate this difference. In a similar study, Chen et al. measured intestinal wall attenuation in a series of patients with mesenteric ischemia to define an arterial and venous contrast ratio compared to a non-contrast phase for affected and non-affected bowel wall in each patient. Decreased ratios were a reliable diagnostic marker for ischemia, however, no comparison was made to patients with other etiologies of bowel wall change, and the protocol relied on a non-contrast scan as a comparator for enhancement [13]. There is a paucity of research on direct measurements of colon wall attenuation in various disease entities and the diagnostic differentiation remains challenging due to mimicked hypoattenuation in differential diagnoses, such as colitis and in patients with liver cirrhosis [14, 21–23]. While risk scores including imaging features for patients with ischemic colitis have been proposed [24], there is no widely adapted diagnostic score combining different imaging features.

This study has several limitations. As patients were preselected for colon wall changes, and in order to ensure a strong correlation with surgical and endoscopic findings, image analysis was performed in a non-blinded setting to the final diagnosis. However, calculations of relative enhancement were only performed after image analysis was completed in all cases. Additionally, a substantial subset of cases was reevaluated with blinding to the final diagnosis to mitigate subconscious bias, showing a strong intra-reader correlation. Analysis of the images for this study took place outside of clinical routine, and the time to place all 12 ROIs per patient was not measured. The readers estimate a range of five to ten minutes per case, varying strongly due to different overall case complexities, additional time would be needed to calculate the averages of ROIs and the relative measurements. This may present a limiting factor implementing such a measurement in clinical routine. Due to the comparative enhancement measurement, patients with pan-colonic wall changes were excluded in the study, introducing a measure of selection bias. This potentially excludes very severe cases of ischemia, where multiple vascular territories are affected, a further limitation in clinical practice as a delay in diagnosis may lead to an unsalvageable situs. Only a subset of patients in the non-ischemia group received a gold-standard diagnostic test, as this was often not clinically necessary, however this may mean, that there are cases of mild ischemia that were never diagnosed as such. A missed diagnosis of ischemia may negatively influence the sensitivity and specificity of the measured cut-off for hypoenhancement. Cases where imaging suspicion was very high, but no confirmatory test was performed were excluded to reduce the number of potential false negative cases. The method used for evaluation of the colonic wall by small ROIs incorporates only a miniscule segment of the bowel wall; a more comprehensive analysis, including larger, non-circular ROIs fitted to the bowel wall or three-dimensional ROIs, could facilitate a more precise measurement of wall attenuation. While the ROIs used were small, correct placement may be difficult in areas of severely thinned bowel wall, and adjacent tissue or luminal contents must be avoided to attain reliable measurements. Conversely, in cases of severe wall edema the ROI may not be able to sufficiently measure enough of the wall thickness for a representative sample. There may be use in adjusting the ROI from a fixed size to a fitted size based on average wall thickness. As advanced segmentation algorithms for CT images become available, a (semi-)automatic evaluation of attenuation along the entire bowel wall may become reality in the future. While intra-reader correlation was strong, inter-reader correlation was only moderate. A retrospective case review between the readers demonstrated the large effect only a slight change in routine placement of the ROI could have, e.g. a tendency to place the ROI slightly more towards the mucosal or serosal side of the bowel wall. It is evident that a more robust multi-reader trial is needed to validate the current method as another tool for ischemia detection, potentially with a joint review of selected cases beforehand in order to harmonize ROI placement and minimize variability. Currently, the proposed method remains experimental and clinical applicability is limited. Analyses of a larger cohort in a multi-center study are needed to potentially grade the severity of ischemia with this method and define a potential relationship between loss of enhancement and interoperative/endoscopic grade of ischemia, as well as potential outcome effects.

A significant difference in relative wall attenuation was observed in patients with ischemia compared to those with other causes of colon wall changes, while relative contrast uptake did not demonstrate definitive differences. This preliminary approach for an objective measurement of bowel wall enhancement shows the potential to be a further tool in the multifaceted imaging diagnosis of colon ischemia.

## Supplementary Information

Below is the link to the electronic supplementary material.

Supplementary Material 1

## Data Availability

The datasets used and/or analyzed during this current study are available from the corresponding author upon reasonable request.

## Abbreviations

AMI: Acute Mesenteric Ischemia
AP: Arterial Phase
AUC: Area Under the Curve
ED: Emergency Department
HU: Hounsfield Units
ICC: Intraclass Correlation Coefficient
ICU: Intensive Care Unit
NOMI: Non–occlusive mesenteric ischemia
ROC: Receiver Operating Characteristic
ROI: Region of Interest
VP: Venous Phase
